# A model for predicting court decisions on child custody

**DOI:** 10.1371/journal.pone.0258993

**Published:** 2021-10-21

**Authors:** José Félix Muñoz Soro, Carlos Serrano-Cinca

**Affiliations:** 1 Aragonese Foundation for Research & Development (ARAID), Zaragoza, Spain; 2 Faculty of Economy and Business, University of Zaragoza, Zaragoza, Spain; University of Kwazulu-Natal, SOUTH AFRICA

## Abstract

Awarding joint or sole custody is of crucial importance for the lives of both the child and the parents. This paper first models the factors explaining a court’s decision to grant child custody and later tests the predictive capacity of the proposed model. We conducted an empirical study using data from 1,884 court rulings, identifying and labeling factual elements, legal principles, and other relevant information. We developed a neural network model that includes eight factual findings, such as the relationship between the parents and their economic resources, the child’s opinion, and the psychological report on the type of custody. We performed a temporal validation using cases later in time than those in the training sample for prediction. Our system predicted the court’s decisions with an accuracy exceeding 85%. We obtained easy-to-apply decision rules with the decision tree technique. The paper contributes by identifying the factors that best predict joint custody, which is useful for parents, lawyers, and prosecutors. Parents would do well to know these findings before venturing into a courtroom.

## Introduction

The most recent data in the US accounted for 2,015,603 marriages and 746,971 divorces annually [1], while in Europe, there were 1,950,935 marriages and 834,068 divorces [2]. After separation or divorce, joint physical custody is increasingly common in many Western societies. This is a parental care arrangement in which a child lives with each parent 25–50% of the time [3]. Joint custody is likely to be beneficial to children on average, which justifies recommending it [4], although the economic repercussions are not negligible and some parents fight for custody to avoid paying child support [5]. However, before engaging in expensive litigation, it would be good for parents to have an idea of how likely it is that they will win the lawsuit. Our paper aims to identify the factual elements that determine a court’s decision to choose joint or sole custody, relate them to the legal principles applied in the judgments, and develop a predictive model capable of forecasting judicial decisions from a set of factual findings.

Our first research question aims to find the factors that explain court rulings on child custody. The best interest of the child principle stands out among the legal principles that influence a court’s decision [6]. Family systems theory argues that a proper custody decision requires an evaluation of the entire family and its relationships [7]. Therefore, legal principles concerning parents, such as the principle of equality between parents and proportionality in responsibility, should be relevant [8]. It is therefore important to know the parents’ relationship and attitude, the parents’ readiness, including the economic resources of both parents, and their previous dedication to childcare. One fact that seems crucial is the parents’ agreement on the type of custody, if any. Among other theories, therapeutic justice justifies support for joint or sole custody depending on what is in the best interest of the child. This theory focuses on the impact of the law on the psychological well-being of individuals, but without privileging therapeutic outcomes over due process or other constitutional and related values [9]. Thus, it is frequent that the child is asked for his or her opinion, while the child’s circumstances and the child’s background are also considered as factual findings that influence the decision on the type of custody [9]. Furthermore, the judge can rely on a psychological report [10]. This research question goes beyond identifying the factual findings and tries to understand the judge’s reasoning and its relationship to the facts. To this end, we developed explanatory models using linear and logistic multivariate regressions.

Already in 1897, Oliver Wendell Holmes, the father of legal realism, claimed that law must be predictive, but one may still wonder to what extent justice is predictable in practice [11]. Our second research question aims to develop a forecasting model. There is extensive literature on legal judgment prediction, for example, on forecasting criminal sentencing decisions [12] and on the prediction of decisions made by the Supreme Court of the United States [13–16]. Explanatory factors for child custody decisions were also studied [10,17,18], but as far as we know, no predictive models have yet been developed to forecast child custody decisions. We applied logistic regression models as benchmarks and other data mining tools with high predictive capacity, such as neural networks. In this research question, we also aim to obtain decisional rules through decision trees. This technique can be very useful not only in predicting court sentences but also in explaining them [15].

We conducted an empirical study with 1,884 Spanish court rulings on child custody. Our models predict the court’s decision with an accuracy exceeding 85%. In the case under analysis (second instance appeals), the justice system agrees with the petitioner only 17.8% of the time. Interestingly, the decision tree detected situations with both extremely high and low probabilities of winning at trial. The latter is a sure loss of money for the litigants and a time-consuming process for the overburdened court system. Widespread use of legal decision support systems would help minimize the asymmetry of information, which is so negative for the justice system [19]. Decision systems such as the one presented in the paper could help alleviate pressure on the justice system, as many parents would avoid going to court and opt for out-of-court settlements.

This study contributes to the literature in many ways. The factors that explain child custody decisions were studied by other authors [17,18,20], but we developed a predictive model, which was tested using temporal validation. This means that the test sample comes from a period after the training sample: this is the appropriate validation method to test predictive results over time [21]. It recalls a real-world situation in which a lawyer estimates the model from the most recent information available and tests the model with new cases. We provide performance measures such as accuracy, sensitivity, and specificity. We provide heuristics in the form of easy-to-apply decision rules, which is a significant practical tool for a lawyer to use to prepare their case for trial. Although court decisions have been analyzed using decision trees [15], no previous research applied decision trees to the study of child custody to the best of our knowledge. Finally, our paper is not limited to an empirical exercise but identifies the underlying legal principles, deepening the causal reasoning behind a judicial decision.

## Literature review

### Legal judgment prediction

Decision-making support systems in the field of justice may take many forms, including computer-assisted legal research [22], expert systems that explain court decisions based on argumentation mining [23], systems for predicting crime [24] and recidivism of juvenile offenders [25], big data tools to help regulators pass appropriate laws by predicting their outcomes [26], and systems that predict judicial decisions [27]. Legal judgment prediction tries to identify factual elements that influenced past court decisions to correctly predict the decisions of new cases for a specified legal problem. As a research field, it has been active since the late 1950s [28], and today, it has great potential due to advances in the natural language processing of judgments [27] and data mining techniques applied to court decision forecasting [15].

Holmes was a pioneer in proposing the predictive theory of law in 1987 [11]. Since then, there have been numerous attempts to predict legal rulings [13–16,28–30] and also outcomes, such as crime outcomes for released defendants [31] and the recidivism of convicts [32]. The most studied topic is that of predicting U.S. Supreme Court rulings [14–16,33].

The predictor variables depend on the chosen theoretical framework. Up to nine judicial decision-making theories were described [34], the most extreme of which are legalism and attitudinalism. Legalism is often regarded as the ‘official theory’ of judicial behavior: judges make decisions exclusively applying the so-called ‘rule of law’ with intellectual rigor. Hence, advocates of legalism use factual findings as explanatory variables, because the judge bases his decision on them [28]. On the other side, advocates of the attitudinal theory argue that judges’ decisions are best explained by personal factors such as emotions, opinions, and political preferences. Some empirical studies incorporated the political preferences of the court and the pressures of interest groups as predictor variables [30]. Other studies derive from psychological theories and even used emotional arousal (measured by a vocal pitch) to predict a jury’s vote [13]. Katz et al. [14] used as many as 240 variables, including chronological variables, case history variables, justice-specific variables, and outcome variables. These last authors used data from the United States Supreme Court Database to forecast 240,000 justice votes over nearly two centuries (1816–2015). Accuracy rates ranged from 71.9% [14] to 75.0% [15,16].

As for the techniques used to predict judicial decisions, the first studies used logistic regression and other multivariate statistical techniques [29]. More recently, studies have used neural network models [35] and techniques based on decision trees [16], including AdaBoosted decision trees [15] and random forest classifiers [14]. Ruger et al. [16] forecasted Supreme Court decisions by comparing two methods: the first was a decision tree model that relied on general case characteristics, while the second was based on a set of predictions made by legal specialists. The statistical model outperformed the experts and was particularly good at forecasting economic activity cases, while the experts did comparatively better in the judicial power cases. It is common to compare the results of the most advanced techniques with those obtained with logistic regression [15] or to compare several techniques with each other [35].

### Joint custody of a child

The Declaration of the Rights of the Child [36] marked the point at which children began to be seen as holders of rights independent of those of their parents, giving substance to the principle of the best interests of the child. This principle governs decisions on custody in cases of separation or divorce, providing the solution that is least traumatic for the child and ensuring that the best conditions for its development should always be sought [6]. However, the principle of the interest of the child is highly indeterminate, and its application depends on multiple factors. Until the 1960s, the ‘tender years’ doctrine predominated in jurisprudence, which considered that there was a biological superiority of the mother over the father, making her preferable for the custody of children [37]. Gradually, positions in favor of shared custody gained acceptance, arguing that it was the modality that, for the child, most resembled the situation before the separation. Joint custody is now a global trend whose antecedent is gender equality, which can be seen in women’s labor participation and fathers’ involvement in childcare [3]. Joint custody currently represents between 10% and 30% of arrangements in Western countries, reaching 35% in Sweden [38] and 38% in Spain [39].

The effects of joint custody on children and parents have been widely studied [40,41]. A recent meta-analysis on 60 studies found that joint custody had better outcomes according to all measures in 34 studies, equal outcomes on some measures, and better outcomes on other measures in 14 studies, with very few cases having worse outcomes [41]. However, these studies have a methodological flaw: they do not come from a randomized control trial, because there will never be an instance of judges assigning the custody of children at random [4]. The expected advantages of shared custody are explained by bonding and monitoring theories [42]. The former argues that parents allow themselves to grow more attached to their children when they do not fear a complete break with them in case of divorce. Monitoring theories ensure that the parents minimize the problem of agency costs, because they know how their financial contributions are spent and assume their commitments responsibly. The type of custody not only impacts the child and the parents but also affects society as a whole. The latter is because the effects of adopting joint custody may include changes in marriage rates, overall fertility, and divorce rates [5].

## Empirical study

### Sample and data

Table 1 describes the variables used in the present study. The dependent variable is the decision made by the court (DEC_JOINT). This is a dummy variable that takes the value 1 if joint custody was decided and 0 in the case of sole custody. What the plaintiff asked for can be considered another dummy dependent variable (RQ_JOINT). The third dependent variable is also a dummy variable that measures whether or not the plaintiff won the lawsuit (WINNER). The independent variables are the factual elements, which deal with the circumstances of the child, the parents, and their relationships [6,8,10,43]. Among the child’s circumstances, the opinions and wishes expressed by the child are usually taken into account (CHILD_OPIN), as well as the psychophysical circumstances of the child (CHILD_PSY) and the child’s adjustment or background (CHILD_ROOT). Concerning the parents, we considered their relationship and attitude towards their obligations (PAR_RELAT), their availability, including schedules and financial resources (PAR_RDNS), their dedication during cohabitation (PAR_DED), and the agreements and conventions between parents (PAR_AGREEM). Finally, we considered the content of the psychosocial report, if any (PSY_REP).

**Table 1:** The variables used in the study and their definitions.

Dependent variable	
**DEC_JOINT**	Dummy variable indicating the type of physical custody decided by the court (1 = joint custody; 0 = sole custody).
**RQ_JOINT**	Dummy variable indicating the type of physical custody requested by the plaintiff (1 = joint custody; 0 = sole custody).
**WINNER**	Dummy variable indicating whether the plaintiff won the trial (1 = won the trial; 0 = lost the trial)
Factual findings	
**PSY_REP**	Net number of occurrences of the psychological report affecting the type of custody. It was obtained by adding up the occurrences in favor of joint custody and subtracting the occurrences in favor of sole custody.
**CHILD_OPIN**	Net number of occurrences of the child’s opinions and wishes expressed affecting the type of custody. It was obtained by adding up the occurrences in favor of joint custody and subtracting the occurrences in favor of sole custody.
**CHILD_PSY**	Net number of occurrences of the child’s psychological circumstances affecting the type of custody. It was obtained by adding up the occurrences in favor of joint custody and subtracting the occurrences in favor of sole custody.
**CHILD_ROOT**	Net number of occurrences of the child’s roots affecting the type of custody. It was obtained by adding up the occurrences in favor of joint custody and subtracting the occurrences in favor of sole custody.
**PAR_RELAT**	Net number of occurrences of the parents’ relationship and attitude affecting the type of custody. It was obtained by adding up the occurrences in favor of joint custody and subtracting the occurrences in favor of sole custody.
**PAR_RDNS**	Net number of occurrences of the parents’ readiness affecting the type of custody. It was obtained by adding up the occurrences in favor of joint custody and subtracting the occurrences in favor of sole custody.
**PAR_DED**	Net number of occurrences of the parents’ previous dedication affecting the type of custody. It was obtained by adding up the occurrences in favor of joint custody and subtracting the occurrences in favor of sole custody.
**PAR_AGREEM**	Net number of occurrences of the parents’ agreements affecting the type of custody. It was obtained by adding up the occurrences in favor of joint custody and subtracting the occurrences in favor of sole custody.
Legal principles	
**BEST_INT**	Net number of occurrences of the ‘best interests of the child’ principle affecting the type of custody. It was obtained by adding up the occurrences in favor of joint custody and subtracting the occurrences in favor of sole custody.
**PAR_EQL**	Net number of occurrences of the ‘parents’ equality’ principle affecting the type of custody. It was obtained by adding up the occurrences in favor of joint custody and subtracting the occurrences in favor of sole custody.
**PROP_RESP**	Net number of occurrences of the ‘proportionality in the responsibilities’ principle affecting the type of custody. It was obtained by adding up the occurrences in favor of joint custody and subtracting the occurrences in favor of sole custody.
**RES_JUD**	Net number of occurrences of the ‘res judicata’ principle affecting the type of custody. It was obtained by adding up the occurrences in favor of joint custody and subtracting the occurrences in favor of sole custody.
Other variables	
**PLAIN_MALE**	Dummy variable indicating the sex of the plaintiff (1 = male; 0 = female).
**JUDGE_MALE**	Dummy variable indicating the sex of the judge (1 = male; 0 = female).
**DATE**	Date of sentence
**FAVOR_JOINT**	Dummy variable indicating whether regional legislation favors or does not favor joint custody (1 = favor; 0 = not in favor)

We considered four legal principles used to understand the judge’s reasoning and its relationship to the aforementioned factual elements [6,8,10,43]. The best interest of the child (BEST_INT) stands out, which, depending on the circumstances of the case, can operate in favor of both sole and joint custody [6]. Other principles include the principle of equality between parents (PAR_EQL) and the principle of proportionality in the assumption of burdens (PROP_RESP), which concerns how each parent should bear the expenses of the children in proportion to their ability to do so [8]. Finally, the principle of res judicata (RES_JUD) was included, which the judge applies when the intention is to modify previously established conditions without a substantial change in circumstances.

The sex of the plaintiff was included as an independent variable (PLAINTIFF_SEX), because this variable was found to be relevant in child custody decisions [44]. Currently, in Spain, mothers obtain the majority of sole custody arrangements, obtaining child custody 58% of the time, while men obtain sole custody 4% of the time and shared custody occurs in the other 38% of cases [39]. The sex of the judge was also included (JUDGE_SEX), because statistically significant differences were found in other contexts [45]; however, we do not expect them to occur in our study.

We also studied the role of territorial legislation. Spain is divided into seventeen autonomous communities, four of which have adopted their own civil legislation on custody. We created a dummy variable (FAVOR_JOINT) that assigns a 1 to rulings issued by courts in territories that established joint custody as preferential (Aragon, Basque Country and Catalonia) and a 0 to the rest of Spain. In Aragon, Law 2/2010 established joint custody as a preferential modality, but Law 6/2019 eliminated this preference and equated both modalities. In the Basque Country, Law 7/2015 established the preference for joint custody, which is still in force. The Civil Code of Catalonia (Law 25/2010) also established the preference for shared custody, although it was more ambiguous in its pronouncements.

**Fig 1:**
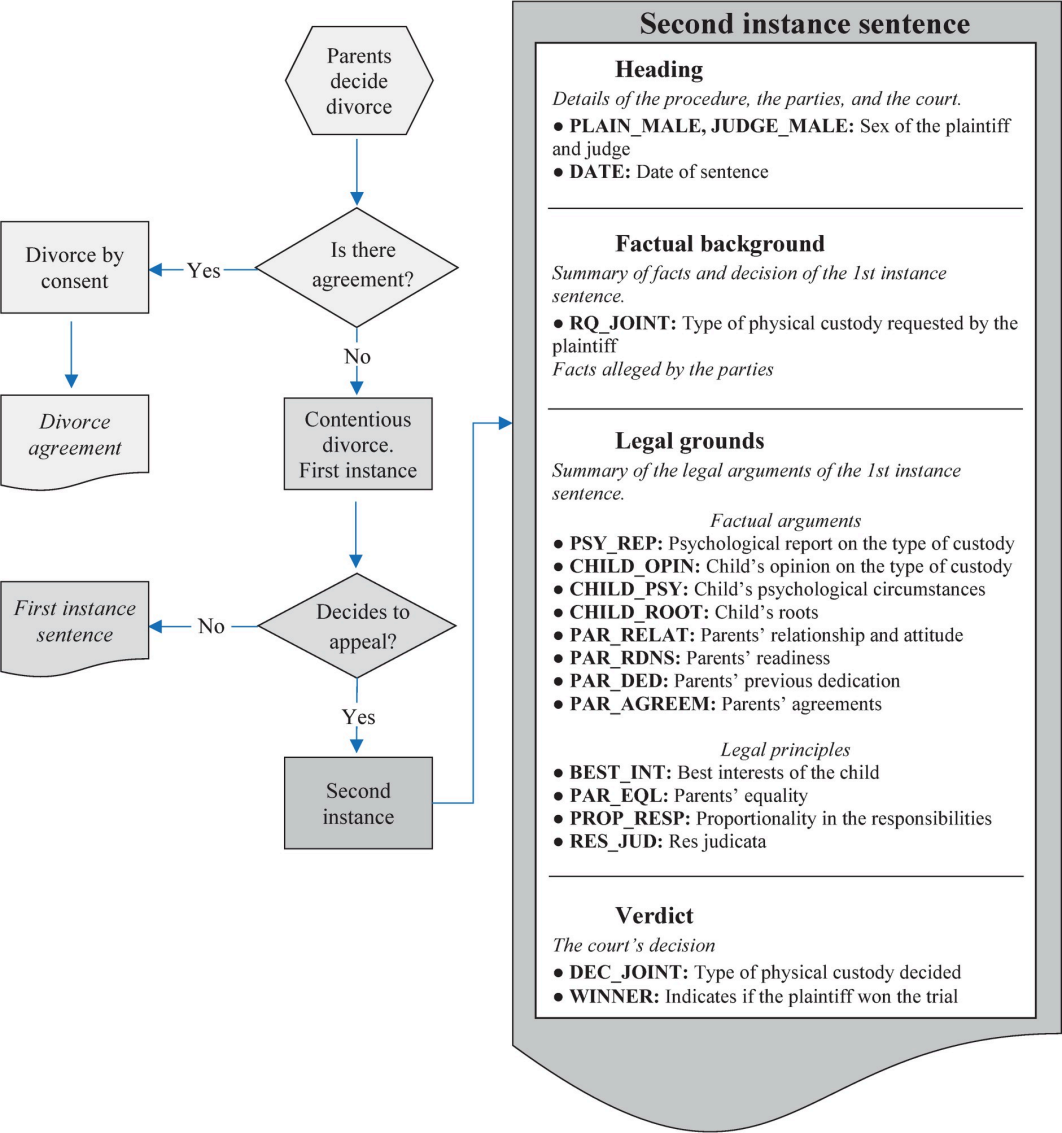
Flowchart of divorce proceedings and contents of a second instance sentence concerning child custody.

The court sentences were taken from the Spanish Judicial Authority Documentation Center (CENDOJ), a body that compiles and disseminates the jurisprudence of the Supreme Court and other Spanish courts. This database is freely accessible to the public. We downloaded 1,884 child custody rulings from June 2016 to June 2020, of which 1,134 (60.2%) were sole custody and 750 were joint custody (39.8%). They are second instance appeal judgments. That is, after a divorce without an agreement, or when one of the parents requested a change in the pre-existing custody modality, the first verdict issued by a judge was not accepted by one of the parties and was appealed. Fig 1 shows a flow diagram of the divorce procedure and the content of the second instance sentence, which is common in both cases.

### Method and procedure

The research team read and labeled the contents of each court sentence, identifying the factual elements and legal principles. Understanding legal language requires expertise in legal matters and two researchers (law graduates) were chosen to label each of the court sentences. This task can be subjective, and the two researchers independently labeled each of the court sentences to minimize bias. Although the criteria were previously agreed upon and the degree of coincidence was high, numerous disagreements arose in the identification of the factual elements and legal principles. Therefore, a third person, the leading researcher, solved the dubious cases. This ensured the quality of the process.

The labeling process was time-consuming and difficult. On average, each court sentence had 2,093 words. On average, we identified 11.46 factual findings and 1.98 legal principles in each sentence. It took about 24 minutes to label each court sentence. Fig 1 also shows the contents of a court sentence on child custody. A court sentence is made up of four parts: (1) the header contains the details of the court, the parties involved, and the professionals who represent and defend them; (2) the factual background explains the factual basis of the decision; (3) the legal grounds contains the legal argument; and (4) the verdict contains the court’s decision. It is important to note that the court may mention some facts alleged by the parties in the factual background, but they may not be taken into account by the court, and hence they must not be labeled. That is, a fact is relevant to the analysis of the argument when it is mentioned in the legal grounds and used as part of the legal argument.

A court sentence may refer to the same factual element several times, sometimes with arguments in favor and sometimes with arguments against. Table 2 shows two examples of phrases for each of the factual elements and legal principles, one favoring joint custody and the other favoring sole custody. Therefore, when labeling each phrase, the meaning was taken into account: if it spoke in favor of joint custody, it was recorded with a positive value, while if it spoke in favor of sole custody, it was recorded with a negative value. Thus, the final value of each variable was obtained by adding up the number of occurrences in favor of joint custody and subtracting the number of occurrences in favor of sole custody. Therefore, the factual findings and legal principles in Table 1 are not dummy variables but quantitative variables.

**Table 2:** Examples of phrases identified in the court sentences for each of the factual elements and legal principles.

Factual findings		
**PSY_REP**	*In favor of joint custody*	The report prepared by the psychosocial technical team concludes by advising the regime of shared custody. *(El informe elaborado por el equipo técnico psicosocial concluye aconsejando el régimen de custodia compartida)*
	*In favor of sole custody*	The psychosocial team’s report considers it necessary and timely for the mother to have custody of her daughters. *(El informe del equipo psicosocial valora necesario y oportuno que la madre tenga la guarda y custodia de sus hijas)*
**CHILD_OPIN**	*In favor of joint custody*	The son has expressed his desire to be able to be equally with both parents. *(El hijo ha manifestado su deseo de poder estar por igual con ambos progenitores)*
	*In favor of sole custody*	The child does not wish to have contact with her father. *(La menor no desea tener contacto con su padre)*
**CHILD_PSY**	*In favor of joint custody*	The upcoming age of majority of the minor advises a last effort in order to relaunch and improve the parent-child relationship through the shared custody regime. *(La cercana mayoría de edad de la menor aconseja un último esfuerzo en orden a relanzar y mejorar la relación paterno-filial a través del régimen de custodia compartida)*
	*In favor of sole custody*	In short, the child is happy and perfectly integrated socially, in their family, and academically, all of which advises at this time maintaining current custody in favor of the mother. *(En definitiva*, *el niño se encuentra feliz y perfectamente integrado social*, *familiar y escolarmente*, *todo lo cual aconseja en este momento mantener la guarda y custodia actual en favor de la madre)*
**CHILD_ROOT**	*In favor of joint custody*	Both minors have an affective bond with both parents and value contact, communication, and staying with both parents positively. *(Ambos menores tienen vinculación afectiva con ambos progenitores y valoran positivamente el contacto*, *comunicación y estancia con ambos)*
	*In favor of sole custody*	The ties established with the mother are closer than those established with the father. *(Son más estrechos los vínculos establecidos con la madre que los que tiene con su padre)*
**PAR_RELAT**	*In favor of joint custody*	It is ruled out that there is a level of conflict between the parents that could be an insurmountable obstacle or inconvenience, so that said shared custody regime can function properly. *(Se descarta que haya un nivel de conflictividad entre los padres que pueda ser un óbice o inconveniente insalvable para que dicho régimen de guarda y custodia compartida pueda funcionar adecuadamente)*
	*In favor of sole custody*	This poor relationship that exists between parents hinders the proper development of joint custody. *(Esta mala relación que existe entre los progenitores dificulta el desarrollo adecuado de la guarda y custodia compartida)*
**PAR_RDNS**	*In favor of joint custody*	The proximity of the domiciles of the litigants also favors the judicial regulation of the alleged shared custody. *(Igualmente propicia la regulación judicial de la pretendida custodia compartida la circunstancia de la proximidad de los domicilios de los litigantes)*
	*In favor of sole custody*	The professional activity of the father is incompatible with shared custody. *(La actividad profesional del padre resulta incompatible con un régimen compartido de guarda y custodia)*
**PAR_DED**	*In favor of joint custody*	Both parents, depending on each parent’s work schedule in each time period, devoted time to the care and attention of their daughter. *(Ambos progenitores*, *en función de los horarios de trabajo de cada uno en cada periodo temporal*, *dedicaron tiempo al cuidado y atención de su hija)*
	*In favor of sole custody*	The mother has been the main caregiver and currently has a job that allows her to take care of the girl. She seems to be the most suitable parent to assign custody of the child. *(Habiendo sido la madre la cuidadora principal y contando en la actualidad con un empleo que le permite hacerse cargo de la niña parece el progenitor más adecuado para asignarle la guarda de la menor)*
**PAR_AGREEM**	*In favor of joint custody*	The weekly shared custody regime is a regime that has been developed by mutual agreement of the parents. *(El régimen de custodia compartida semanal es un régimen que se ha venido desarrollando*, *por mutuo acuerdo de los progenitores)*
	*In favor of sole custody*	Deciding by mutual agreement that the daughters would remain in the custody of the father. *(Decidiendo de mutuo acuerdo que las hijas quedarían bajo la guarda y custodia del padre)*
*Legal principles*		
**BEST_INT**	*In favor of joint custody*	In the interests of the child, a shared custody regime would be advisable. *(En interés del menor sería aconsejable un régimen de custodia compartida)*
	*In favor of sole custody*	There are no objective reasons that guarantee that the child’s interest is to a greater extent protected through joint custody. *(No existen razones objetivas que avalen que el interés del menor esté en mayor medida protegido a través de una custodia compartida)*
**PAR_EQL**	*In favor of joint custody*	To have an equal share in the development and growth of their children undoubtedly seems to be the most beneficial for them as well. *(Participar en igualdad de condiciones en el desarrollo y crecimiento de sus hijos*, *lo que sin duda parece también lo más beneficioso para ellos)*
	*In favor of sole custody*	The interest of the child must prevail over the principle of equal rights between parents. *(El interés del menor debe prevalecer sobre el principio de igualdad de derechos entre los progenitores)*
**PROP_RESP**	*In favor of joint custody*	Each parent shall bear the cost of food, lodging, and clothing for the children during their custodial shift. *(Cada progenitor asumirá los gastos de alimentación*, *alojamiento y vestido de los menores durante su turno de custodia)*
	*In favor of sole custody*	The amount of maintenance must be proportional to the means at the disposal of the maintainer, taking into account the financial capacity of the non-custodial parent. *(La cuantía de la pensión de alimentos debe ajustarse a criterios de proporcionalidad entre los medios con los que cuenta el alimentante y las necesidades del alimentista*, *para lo que se hace preciso tener en cuenta la capacidad económica del progenitor no custodio)*
**RES_JUD**	*In favor of joint custody*	In short, the existence of a change in circumstances that justifies the alteration of the shared custody regime established in the previous judgment cannot be admitted. *(En definitiva*, *no puede admitirse la existencia de un cambio de circunstancias que justifique la alteración del régimen de guarda y custodia compartida fijado en la sentencia anterior)*
	*In favor of sole custody*	Submission to consideration of the opportunity for a shared custody regime cannot be reconsidered unless there is a substantial change in circumstances. *(No cabe replantear el sometimiento a consideración de la oportunidad de establecimiento de un régimen de custodia compartida*, *si no se da una alteración sustancial de las circunstancias)*

In italics, the original in Spanish.

## Results

### Factual elements and legal principals associated with each court decision

Table 3 provides an overview of the summary statistics for each independent variable for the two groups of court sentences (joint and sole custody) according to the judge’s decision (DEC_JOINT). The table reports the mean, standard deviation, and minimum and maximum values for both groups. An independent samples t-test was conducted to determine if the composition of the factual elements and legal principles differed for the two possible decisions of the court (joint and sole). As expected, a court sentence that decides on joint custody includes on average more phrases that refer to factual elements that favor joint custody than phrases that favor sole custody. The differences are statistically significant for all factual elements and legal principles.

**Table 3:** Sample descriptives using t-test for equality of means.

	Decision: sole custody DEC_JOINT = 0; N = 1,134				Decision: joint custody DEC_JOINT = 1; N = 750				
	*Mean*	*St dev*	*Min*	*Max*	*Mean*	*St dev*	*Min*	*Max*	*t-test*
**PSY_REP**	-0.183	0.881	-5	3	0.196	0.874	-3	4	-9.178***
**CHILD_OPIN**	-0.373	1.266	-10	8	0.261	0.935	-3	5	-11.765***
**CHILD_PSY**	-0.782	1.844	-16	10	0.825	1.310	-4	7	-20.669***
**CHILD_ROOT**	-0.101	1.025	-6	7	0.413	1.094	-3	8	-10.390***
**PAR_RELAT**	-0.594	2.999	-14	12	3.012	2.991	-7	17	-25.574***
**PAR_RDNS**	-1.331	3.071	-16	12	1.881	3.360	-9	16	-21.398***
**PAR_DED**	-0.400	1.149	-6	4	-0.052	0.823	-4	4	-7.174***
**PAR_AGREEM**	-0.110	0.544	-3	2	0.000	0.482	-3	4	-4.502***
**BEST_INT**	-1.038	2.243	-11	13	1.559	2.094	-11	14	-25.250***
**PAR_EQL**	-0.023	0.213	-3	2	0.077	0.318	-1	3	-8.203***
**PROP_RESP**	-0.137	0.568	-3	2	0.020	0.430	-2	2	-6.430***
**RES_JUD**	-0.057	0.346	-3	3	0.037	0.239	-1	3	-6.535***

The two groups differentiate the type of custody decided by the court (DEC_JOINT).

*** significant at the 1% level.

Table 4 is similar to the previous table but uses the request for joint custody and sole custody as a grouping variable (RQ_JOINT). It should be remembered that these are second instance appeal proceedings; therefore, they were already subject to a judgment, and the plaintiff is asking for a new trial. The results show a negative relationship between the petition and the factual elements. That means that petitions for joint custody include on average more labels referring to factual elements that favor sole custody than labels favoring joint custody. Again, the differences are statistically significant for all variables.

**Table 4:** Sample descriptives using t-test for equality of means.

	Request: sole custody RQ_JOINT = 0; N = 853				Request: joint custody RQ_JOINT = 1; N = 1,031				
	*Mean*	*St dev*	*Min*	*Max*	*Mean*	*St dev*	*Min*	*Max*	*t-test*
**PSY_REP**	0.155	0.881	-5	4	-0.187	0.882	-5	3	8.381***
**CHILD_OPIN**	0.124	1.172	-7	8	-0.323	1.161	-10	5	8.288***
**CHILD_PSY**	0.574	1.745	-16	10	-0.735	1.681	-9	5	16.544***
**CHILD_ROOT**	0.328	1.123	-4	7	-0.082	1.011	-6	8	8.348***
**PAR_RELAT**	1.986	3.124	-14	14	-0.106	3.471	-14	17	13.619***
**PAR_RDNS**	0.809	3.419	-16	14	-0.764	3.509	-16	16	9.799***
**PAR_DED**	-0.045	0.975	-5	4	-0.441	1.068	-6	4	8.348***
**PAR_AGREEM**	0.036	0.475	-2	4	-0.151	0.545	-3	2	7.879***
**BEST_INT**	1.081	2.233	-11	14	-0.902	2.402	-11	13	18.409***
**PAR_EQL**	0.036	0.256	-2	3	0.001	0.270	-3	2	2.898***
**PROP_RESP**	-0.021	0.408	-3	2	-0.118	0.599	-3	2	4.031***
**RES_JUD**	0.035	0.272	-1	3	-0.065	0.334	-3	1	7.044***

The two groups differentiate the type of custody requested by the plaintiff (RQ_JOINT).

*** significant at the 1% level.

### Relationships between requests, court decisions, sex of the plaintiff and sex of the judge

Table 5 presents contingency tables that show the relationships between requests, court decisions, the sex of the plaintiff and the sex of the judge. Contingency tables were analyzed statistically using Pearson’s Chi-square test statistics. The association was estimated with odds ratios (OR) and their respective 95% confidence intervals. Panel A in Table 5 presents the relationship between what was demanded and success in winning or losing the trial. Plaintiffs only win an average of 17.8% of child custody trials. Those who request joint custody are 1.74 times more likely to win than those who request sole custody. The results are statistically significant.

**Table 5:** Contingency tables, Chi-square test and odds ratios for categorical variables.

**Panel A** N (% column) (% row)	**WINNER = 1**	**WINNER = 0**	**TOTAL**	**Odds Ratio for WINNER = 1 (RQ_JOINT (1/0)) [95% confidence interval]**	**Pearson’s χ** ^**2**^
RQ_JOINT = 1	220 (65.7%) (21.3%)	811 (52.4%) (78.7%)	1,031 (54.7%) (100%)	1.74 [1.36–2.23]	19.709***
RQ_JOINT = 0	115 (34.3) (13.5%)	738 (47.6%) (86.5%)	853 (45.3%) (100%)		
	335 (100%) (17.8%)	1549 (100%) (82.2%)	1,884		
**Panel B** N (% column) (% row)	**RQ_JOINT = 0**	**RQ_JOINT = 1**	**TOTAL**	**Odds Ratio for RQ_JOINT = 0 (PLAIN_MALE(0/1)) [95% confidence interval]**	**Pearson’s χ** ^**2**^ ** **
PLAIN_MALE = 0	651 (76.3%) (86.5%)	102 (9.9%) (13.5%)	753 (40.0%) (100%)	29.35 [22.67–38.00]	858.423***
PLAIN_MALE = 1	202 (23.7%) (17.9%)	929 (90.1%) (82.1%)	1,131 (60.0%) (100%)		
	853 (100%) (45.3%)	1,031 (100%) (54.7%)	1,884		
**Panel C** N (% column) (% row)	**WINNER = 1**	**WINNER = 0**	**TOTAL**	**Odds Ratio for WINNER = 1 (PLAIN_MALE (1/0)) [95% confidence interval]**	**Pearson’s χ** ^**2**^ ** **
PLAIN_MALE = 1	227 (67.8%) (20.1%)	904 (58.4%) (79.9%)	1,131 (60.0%) (100%)	1.50 [1.167–1.926]	10.145***
PLAIN_MALE = 0	108 (32.2%) (14.3%)	645 (41.6%) (85.7%)	753 (40.0%) (100%)		
	335 (100%) (17.8%)	1,549 (100%) (82.2%)	1,884		
**Panel D** Plaintiff = male N (% column) (% row)	**WINNER = 1**	**WINNER = 0**	**TOTAL**	**Odds Ratio for WINNER = 1 (RQ_JOINT (1/0)) [95% confidence interval]**	**Pearson’s χ** ^**2**^ ** **
RQ_JOINT = 1	204 (89.9%) (22.0%)	725 (80.2%) (78.0%)	929 (82.1%) (100%)	2.19 [1.38–3.47]	11.562***
RQ_JOINT = 0	23 (10.1%) (11.4%)	179 (19.8%) (88.6%)	202 (17.9%) (100%)		
	227 (100%) (20.1%)	904 (100%) (79.9%)	1,131		
**Panel E** Plaintiff = female N (% column) (% row)	**WINNER = 1**	**WINNER = 0**	**TOTAL**	**Odds Ratio for WINNER = 1 (RQ_JOINT (1/0)) [95% confidence interval]**	**Pearson’s χ** ^**2**^ ** **
RQ_JOINT = 1	16 (14.8%) (15.7%)	86 (13.3%) (84.3%)	102 (13.5%) (100%)	1.13 [0.63–2.01]	0.173
RQ_JOINT = 0	92 (85.2%) (14.1%)	559 (86.7%) (85.9%)	651 (86.5%) (100%)		
	108 (100%) (14.3%)	645 (100%) (85.7%)	753		
**Panel F** N (% column) (% row)	**DEC_JOINT = 1**	**DEC_JOINT = 0**	**TOTAL**	**Odds Ratio for DEC_JOINT = 1 (JUDGE_ML (1/0)) [95% confidence interval]**	**Pearson’s χ** ^**2**^ ** **
JUDGE_ML = 1	475 (63.7%) (39.8%)	719 (63.2%) (60.2%)	1,194 (63.4%) (100%)	1.02 [0.84–1.24]	0.047
JUDGE_ML = 0	271 (36.3%) (39.3%)	419 (36.8%) (60.7%)	690 (36.6%) (100%)		
	746 (100%) (39.6%)	1,138 (100%) (60.4%)	1,884		
**Panel G** N (% column) (% row)	**DEC_JOINT = 1**	**DEC_JOINT = 0**	**TOTAL**	**Odds Ratio for DEC_JOINT = 1 (FAVOR_JOINT (1/0)) [95% confidence interval]**	**Pearson’s χ** ^**2**^ ** **
FAVOR_JOINT = 1	174 (23.4%) (37.9%)	285 (25.0%) (62.1%)	459 (24.4%) (100%)	0.91 [0.74–1.13]	0.679
FAVOR_JOINT = 0	571 (76.6%) (40.1%)	854 (75.0%) (59.9%)	1,425 (75.6%) (100%)		
	745 (100%) (39.5%)	1,139 (100%) (60.5%)	1,884		
**Panel H** N (% column) (% row)	**WINNER = 1**	**WINNER = 0**	**TOTAL**	**Odds Ratio for WINNER = 1 (FAVOR_JOINT (1/0)) [95% confidence interval]**	**Pearson’s χ** ^**2**^ ** **
FAVOR_JOINT = 1	105 (31.3%) (22.9%)	354 (22.9%) (77.1%)	459 (24.4%) (100%)	1.54 [1.19–1.99]	10.773***
FAVOR_JOINT = 0	230 (68.7%) (16.1%)	1,195 (77.1%) (83.9%)	1,425 (75.6%) (100%)		
	335 (100%) (17.8%)	1,549 (100%) (82.2%)	1,884		

*** significant at the 1% level.

Panel B in Table 5 presents the relationship between the sex of the appellant and the claims. Most men request joint custody, while most women request sole custody. Female plaintiffs are 29.35 times more likely to request sole custody than joint custody. The differences are very large and statistically significant. In the first instance in Spanish courts, the most frequent rulings are those that grant joint custody to the mother, which partly explains why it is men who appeal in the second instance, and why they are asking for joint physical custody. Panel C in Table 5 presents the relationship between the sex of the appellant and winning or losing the trial. Male plaintiffs are 1.5 times more likely to win than female plaintiffs. The differences are statistically significant. It is, therefore, appropriate to differentiate between the requests made by men and women, so we split the sample into two subsamples, according to the sex of the applicant. Panel D in Table 5 presents the relationship between petitions and winning or losing the trial when the plaintiff is a man. The most common situation is for a man to ask for shared custody, which accounted for 929 cases out of the full sample of 1,884 court sentences (49.3%). It is unusual for a man to ask for individual custody; they only accounted for 10.7% of the full sample. Males requesting joint custody are 2.19 times more likely to win than those requesting sole custody. The differences are statistically significant. Panel E in Table 5 presents the relationship between petitions and winning or losing the trial when the plaintiff is a woman. There is no significant relationship between petitions and winning or losing the trial when the plaintiff is a woman. Panel F in Table 5 presents the relationship between the sex of the judge and court decisions. There were no statistically significant differences between court decisions made by judges of different sexes.

Panel G relates the courts’ decision (DEC_JOINT) to the existence or not of territorial legislation in favor of joint custody (FAVOR_JOINT). There are no statistically significant differences. Panel H relates the plaintiff’s winning the case (WINNER) to the existence or not of territorial legislation in favor of joint custody (FAVOR_JOINT). Plaintiffs from territories whose legislation favors joint custody are 1.54 times more likely to win the trial than those from territories whose legislation does not favor joint custody. The differences are statistically significant. The rationale is that establishing by law a preferential modality increases legal certainty, hence the chances for success are low in second appeals.

### Relationships between legal principles and factual findings

In the following, we seek to relate the factual findings to legal principles. Legal reasoning follows several steps that can be simplified as follows: identify the issue and the applicable law; analyze and synthesize the legal rules and principles governing the issue; investigate the relevant facts and apply the rule to the facts to obtain the outcome [46]. Legal argumentation encompasses the justification of legal decisions–that is, how conclusions can be reached through logical reasoning. The doctrine of *stare decisis* states that cases that have similar facts should receive similar decisions, thus legal practitioners need to identify such facts and principles in precedent cases, which is a time-consuming task [47]. Table 6 relates factual elements to legal principles through multivariate linear regressions.

**Table 6:** Multivariate linear regressions relating factual elements to legal principles.

	*PSY_REP (Model 1)*	*CHILD_OPIN (Model 2)*	*CHILD_PSY (Model 3)*	*CHILD_ROOT (Model 4)*	*PAR_RELAT (Model 5)*	*PAR_RDNS (Model 6)*	*PAR_DED (Model 7)*	*PAR_AGREEM (Model 8)*	*VIF*
*BEST_INT*	0.054***	0.095***	0.016***	0.090***	0.468***	0.413***	0.084***	0.016***	1.07
*PAR_EQL*	0.057	0.016	0.150**	0.069	0.725**	0.474	-0.091	-0.051	1.06
*PROP_RESP*	0.087**	0.123**	0.074***	0.075	0.506***	1.376***	0.108**	0.014	1.01
*RES_JUD*	0.066	0.084	0.125***	0.118	0.430*	0.369	-0.016	0.113***	1.02
*Constant*	-0.025	-0.110***	0.039***	0.111***	0.877***	0.051	-0.252***	-0.062***	
*Adjusted R* ^*2*^	0.028	0.046	0.158	0.050	0.141	0.147	0.042	0.010	
*F-statistic*	14.390***	23.555***	89.192***	25.859***	78.437***	82.332***	21.869***	5.760***	
*Durbin Watson statistic*	1.913	1.993	1.905	1.980	1.943	1.950	1.962	1.910	
*White’s Chi*^*2*^ *test*	5.96	8.76	9.42	10.37	23.46	6.56	14.72	17.34	
*Shapiro-Wilk W test*	0.825***	0.816***	0.945 ***	0. 838***	0. 983***	0.974***	0.842***	0.591***	
*N*	1,884	1,884	1,884	1,884	1,884	1,884	1,884	1,884	

VIF stands for variance inflation factor.

* significant at 10% level

** significant at 5% level

*** significant at 1% level.

The assumptions of multicollinearity [48], linearity, no auto-correlation [49], and homoscedasticity [50] were all checked and found to be within acceptable thresholds. While there was some deviation from normality, the sample size is large enough to consider the deviation not to have a serious effect on the results [51–53].

The first model uses PSY_REP as a dependent variable and the four legal principles as independent variables. A significant regression equation was found (F(4,1879) = 14.390, p < 0.001), but it had a low adjusted R^2^ of 0.028. The other models also found significant regression equations, with similar values of goodness-of-fit, ranging from 0.010 to 0.158. The only principle with significant coefficients in all eight models is the best interest of the child (BEST_INT). Let us focus on model 6. When the parents’ readiness (PAR_RDNS) factual finding arises, the most frequent argumentation refers to the proportionality in the responsibilities principle (PROP_RESP) and the best interest principle (BEST_INT). Given a fact related to parents’ readiness (i.e. financial resources of both parents, their previous dedication to childcare, the proximity of the domiciles of the litigants, or their professional activity) the judgment refers particularly to the proportionality in the responsibilities principle in addition to the omnipresent best interest of the child principle. Both legal principles were significant determinants of parents’ readiness, but the beta coefficient for the proportionality in the responsibilities principle (1.376) was notably larger than that for the best interests of the child principle (0.413). For example, if the litigants live nearby, it seems quite reasonable for the party seeking shared custody to argue that this factual finding (on parents’ readiness) favors joint custody, alluding to the principle of shared responsibility–always in the best interests of the child. Similar associations can be identified in the remaining facts and legal principles. The parents’ equality principle (PAR_EQL) exhibits a significant relationship with the child’s psychological circumstances (CHILD_PSY), and the res judicata principle (RES_JUD) has a significant relationship with the parents’ agreements (PAR_AGREEM).

### Predicting court decisions

Our second research question was about predicting court decisions. Table 7 shows the results of a logistic regression whose dependent variable is the court decision on custody type (DEC_JOINT). When developing a predictive model, it is necessary to validate it–that is, to evaluate its performance by testing how well the model predicts the dependent variable in the presence of new cases [54,55]. External validation in which the forecasting capacity is tested using a different sample from another time period (temporal validation) was applied. The training sample includes 942 court rulings from June 2016 to May 2019, and the test sample includes 942 court rulings from May 2019 to June 2020.

**Table 7:** Logistic regression analysis and neural network models for predicting court decisions on custody (DEC_JOINT).

*Dependent variable DEC_JOINT*	*Univariate logistic regression (Model 1)*		*Univariate logistic regression (Model 2)*		*Univariate logistic regression (Model 3)*		*Univariate logistic regression (Model 4)*		*Univariate logistic regression (Model 5)*		*Univariate logistic regression (Model 6)*	
*PSY_REP*	0.542***											
*CHILD_OPIN*			0.605***									
*CHILD_PSY*					0.804***							
*CHILD_ROOT*							0.480***					
*PAR_RELAT*									0.419***			
*PAR_RDNS*											0.367***	
*PAR_DED*												
*PAR_AGREEM*												
*PLAIN_MALE*												
*Constant*	-0.425***		-0.367***		-0.430***		-0.481***		-0.916***		-0.517***	
*R2 Nagelkerke*	0.045		0.078		0.217		0.052		0.249		0.206	
*-2 Log likelihood*	1223.113		1189.502		1036.307		1216.061		996.215		1049.120	
*Train sample (N obs*. *= 942)*												
*Confusion matrix*	513	54	530	37	497	70	537	30	497	70	504	63
	304	71	305	70	195	180	329	46	187	188	183	192
*Accuracy (%)*	62.0%		63.7%		71.9%		61.9%		72.7%		73.9%	
*True negative rate (%)*	90.5%		93.5%		87.7%		94.7%		87.7%		88.9%	
*True positive rate (%)*	18.9%		18.7%		48.0%		12.3%		50.1%		51.2%	
*Area under ROC curve (AUC)*	0.591		0.640		0.797		0.620		0.812		0.772	
*Test sample (N obs*. *= 942)*												
*Confusion matrix*	524	43	535	32	491	76	543	24	509	58	492	75
	300	75	305	70	181	194	321	54	169	206	197	178
*Accuracy (%)*	63.6%		64.2%		72.7%		63.4%		75.9%		71.1%	
*True negative rate (%)*	92.4%		94.4%		86.6%		95.8%		89.8%		86.8%	
*True positive rate (%)*	20.0%		18.7%		51.7%		14.4%		54.9%		47.5%	
*Area under ROC curve (AUC)*	0.607		0.628		0.783		0.626		0.830		0.766	

*Dependent variable DEC_JOINT*	*Univariate logistic regression (Model 7)*		*Univariate logistic regression (Model 8)*		*Univariate logistic regression (Model 9)*		*Multivariate logistic regression (Model 10)*		*Multilayer Perceptron Neural Network (Model 11)*		*Radial Basis Function Network (Model 12)*	
*PSY_REP*							0.153		37.9%		59.5%	
*CHILD_OPIN*							0.242**		89.9%		65.2%	
*CHILD_PSY*							0.361***		99.2%		84.4%	
*CHILD_ROOT*							-0.079		29.4%		72.3%	
*PAR_RELAT*							0.255***		100.0%		100.0%	
*PAR_RDNS*							0.233***		62.3%		99.3%	
*PAR_DED*	0.266***						-0.133		43.5%		52.4%	
*PAR_AGREEM*			0.430***				0.251		41.0%		36.6%	
*PLAIN_MALE*					-1.847***		-1.419***		24.7%		63.0%	
*Constant*	0.353***		-0.386***		0.664***		0.026					
*R2 Nagelkerke*	0.018		0.010		0.166		0.563					
*-2 Log likelihood*	1249.787		1256.908		1095.782		759.194					
*Train sample (N obs*. *= 942)*												
*Confusion matrix*	545	22	550	17	444	123	489	78	504	63	491	76
	366	9	355	20	136	239	86	289	44	331	50	325
*Accuracy (%)*	58.8%		60.5%		72.5%		82.6%		88.6%		86.6%	
*True negative rate (%)*	96.1%		97.0%		78.3%		86.2%		88.9%		86.7%	
*True positive rate (%)*	2.4%		5.3%		63.7%		77.1%		88.3%		86.6%	
*Area under ROC curve (AUC)*	0.569		0.530		0.710		0.902		0.929		0.924	
*Test sample (N obs*. *= 942)*												
*Confusion matrix*	554	13	544	23	420	147	491	76	489	78	480	87
	361	14	348	27	1315	244	81	294	50	325	58	317
*Accuracy (%)*	58.8%		60.6%		70.5%		83.3%		86.4%		84.6%	
*True negative rate (%)*	96.1%		95.9%		74.1%		86.6%		86.2%		84.7%	
*True positive rate (%)*	2.4%		7.2%		65.1%		78.4%		86.7%		84.5%	
*Area under ROC curve (AUC)*	0.593		0.538		0.696		0.896		0.918		0.910	

Confusion matrix	
*True negative*	*Type I error*
*Type II error*	*True positive*

Training sample comprises 942 court sentences, where 375 are joint custody and 567 are sole custody, using data from June 2016 to mid-May 2019. Test sample comprises 942 sentences, where 375 are joint custody and 567 are sole custody, using data from mid-May 2019 to June 2020. True negative rate = 1—Type 1 error rate. True positive rate = 1—Type II error rate.

Models 1 through 9 are univariate logistic regression analysis, showing B coefficients and significance levels. Model 10 is a multivariate logistic regression. Model 11 is a multilayer perceptron neural network. Model 12 is a radial basis function neural network. The standardized importance of each variable is shown in both neural network models.

* significant at 10% level

** significant at 5% level

*** significant at 1% level.

We designed the research to be a real-world study in which a law firm wants to develop a predictive model, taking May 2019 as a starting point. The team of lawyers would take the information available on that date, develop the model, and make predictions as new sentences appear, while being able to calculate various performance measures for the model. We used accuracy, true positive rate (sensitivity), true negative rate (specificity), and the area under the receiver operating characteristic curve (AUC) as performance measures. The first eight models are univariate logistic regressions that use each of the factual elements as an explanatory variable. Model 9 uses the sex of the plaintiff as an explanatory variable. The accuracy of univariate models ranges from 58.8% to 75.9%. The independent variable that shows the greatest predictive ability is the relationship between parents (PAR_RELAT). Although the predictive power of this variable is remarkable, it presents slightly unbalanced values in terms of the percentages of type I and type II errors. Model 10 is a full model with all nine independent variables and has an accuracy rate of 83.3%. The percentages of type I and type II errors are fairly balanced.

Table 7 also shows the results of training two neural network models. Model 11 is a multilayer perceptron with a hidden layer and sigmoid activation functions. Model 12 is a radial base function network. The accuracy increased to 86.4% and 84.6%, respectively. The percentages of type I and type II errors are fully balanced. The normalized importance of each variable is shown in both neural network models, and the results coincide with those obtained in the logistic regression.

Table 5 showed that the probability of losing the trial was much higher than that of winning it. This encourages research into decisional rules that may be valuable, which was also the subject of the second research question. We used the Chi-squared Automatic Interaction Detection (CHAID) decision tree to get rules [56]. Table 8 summarizes the results. Again, we divided the sample into training data for the development of the model (n = 942) and testing data for the validation of the model (n = 942). Node 1 of Table 8 shows that 60% of the cases are men who go to trial, and the test sample indicates that they have a probability of winning of 19.6%. Specifically, there are 108 winning cases out of 942, which is 11.5% of the test sample. The probability of winning rises to 21.5% if the man requests shared custody (Node 2). Node 3 provides a winning strategy, as it represents men who request joint custody and have an excellent relationship with their partner (PAR_RELAT); sentences refer to this positive relationship more than 4 times on average. Then, the probability of winning rises to 89.1%. 4.9% of cases meet these characteristics. The probability of winning even increases to 91.3% if the psychophysical circumstances of the child (CHILD_PSY) favor shared custody (Node 4). On the contrary, a man who applies for joint custody and has a bad relationship accompanied by child circumstances that are unfavorable for joint custody has a 99.4% chance of losing at trial (Node 5). This is a frequent scenario, with 349 cases (18.5% of the sample), and only on 2 occasions did the judge grant joint custody. Women who file for joint custody have a small (16.9%) chance of getting it (Node 10), which increases to 80% if the parental relationship is good and the child’s circumstances recommend it, although this only happens 0.9% of the time. If a woman requests individual custody, she will usually lose her case, with a probability of 86.7% (Node 13). This situation accounts for 34.6% of all trials.

**Table 8:** Decision rules for the prediction of the trial outcome from the CHAID algorithm showing winning and losing strategies.

Node	Sex	Request	Rule	Cases (N = 1,884)	Trial outcome	Train (N = 942)		Test (N = 942)	
						Probability	N (%)	Probability	N (%)
1	Male		PLAIN_MALE = 1	1,131 (60%)	Win **Lose**	20.5% 79.5%	119 (12.6%) 461 (48.9%)	19.6% 80.4%	108 (11.5%) 443 (47.0%)
2	Male	Joint-custody	PLAIN_MALE = 1 AND RQ_JOINT = 1	929 (49.3%)	Win **Lose**	22.4% 77.6%	106 (11.3%) 368 (39.1%)	21.5% 78.5%	98 (10.4%) 357 (37.9%)
3	Male	Joint-custody	PLAIN_MALE = 1 AND RQ_JOINT = 1 AND PAR_RELAT>4	92 (4.9%)	**Win** Lose	89.1% 10.9%	41 (4.4%) 5 (0.5%)	89.1% 10.9%	41 (4.4%) 5 (0.5%)
4	Male	Joint-custody	PLAIN_MALE = 1 AND RQ_JOINT = 1 AND 1<PAR_RELAT≤4 AND CHILD_PSY>0	47 (2.5%)	**Win** Lose	91.7% 8.3%	22 (2.3%) 2 (0.2%)	91.3% 8.7%	21 (2.2%) 2 (0.2%)
5	Male	Joint-custody	PLAIN_MALE = 1 AND RQ_JOINT = 1 AND PAR_RELAT≤0 AND CHILD_PSY≤-1	349 (18.5%)	Win **Lose**	0.6% 99.4%	1 (0.1%) 173 (18.4%)	0.6% 99.4%	1 (0.1%) 174 (18.5%)
6	Male	Sole-custody	PLAIN_MALE = 1 AND RQ_JOINT = 0	202 (10.7%)	Win **Lose**	12.3% 87.7%	13 (1.4%) 93 (9.9%)	10.4% 89.6%	10 (1.1%) 86 (9.1%)
7	Male	Sole-custody	PLAIN_MALE = 1 AND RQ_JOINT = 0 AND CHILD_PSY>-1	171 (9.1%)	Win **Lose**	3.4% 96.6%	3 (0.3%) 86 (9.1%)	2.4% 97.6%	2 (0.2%) 80 (8.5%)
8	Male	Sole-custody	PLAIN_MALE = 1 AND RQ_JOINT = 0 AND CHILD_PSY≤-1	31 (1.6%)	**Win** Lose	58.8% 41.2%	10 (1.1%) 7 (0.7%)	57.1% 42.9%	8 (0.8%) 6 (0.6%)
9	Female		PLAIN_MALE = 0	753 (40.0)	Win **Lose**	12.2% 87.8%	44 (4.7%) 318 (33.8%)	16.4% 83.6%	64 (6.8%) 327 (34.7%)
10	Female	Joint-custody	PLAIN_MALE = 0 AND RQ_JOINT = 1	102 (5.4%)	Win **Lose**	14.0% 86.0%	6 (0.6%) 37 (3.9%)	16.9% 86.1%	10 (1.1%) 49 (5.2%)
11	Female	Joint-custody	PLAIN_MALE = 0 AND RQ_JOINT = 1 AND CHILD_PSY>-1 AND PAR_RELAT>1	17 (0.9%)	**Win** Lose	85.7% 14.3%	6 (0.6%) 1 (0.1%)	80.0% 20.0%	8 (0.8%) 2 (0.2%)
12	Female	Joint-custody	PLAIN_MALE = 0 AND RQ_JOINT = 1 AND CHILD_PSY≤-1	49 (2.6%)	Win **Lose**	0.0% 100.0%	0 (0.0%) 24 (2.5%)	0.0% 100.0%	0 (0.0%) 25 (2.7%)
13	Female	Sole-custody	PLAIN_MALE = 0 AND RQ_JOINT = 0	651 (34.6%)	Win **Lose**	11.9% 88.1%	38 (4.0%) 281 (29.8%)	16.3% 86.7%	54 (5.7%) 278 (29.5%)
14	Female	Sole-custody	PLAIN_MALE = 0 AND RQ_JOINT = 0 AND CHILD_PSY≤-1	84 (4.5%)	**Win** Lose	77.1% 22.9%	27 (2.9%) 8 (0.8%)	71.4% 28.6%	35 (3.7%) 14 (1.5%)
15	Female	Sole-custody	PLAIN_MALE = 0 AND RQ_JOINT = 0 AND CHILD_PSY≤-1 AND PAR_RELAT≤0	64 (3.4%)	**Win** Lose	88.5% 11.5%	23 (2.4%) 3 (0.3%)	78.9% 21.1%	30 (3.2%) 8 (0.8%)

Growing method: Exhaustive CHAID. Accuracy of the test (90.7%), true negative rate (93.9%), and true positive rate (77.8%).

## Discussion and conclusion

This paper shows that it is possible to explain and predict court decisions on child custody from a set of factual findings, with a success rate of over 85%. The first research question studied the factors that explain the decision to grant sole or joint custody. Our empirical study was conducted with second instance appeal judgments. We found that all factual elements hypothesized in our model are considered by the judge, whether they are related to the child, the parents, or the psychological report. The relationship and attitudes of the parents and the psychophysical circumstances of the child are the two factual elements that the judge takes most into account when deciding the type of custody. This is very much in line with the family systems theory [7]. The best interest of the child is the only principle with significant coefficients in all regression models. This principle stands out among the legal principles that justify the decision [44], which is supported by the theory of therapeutic justice and most of the explanatory theories of court decisions. In the cases analyzed, the sex of the applicant is very important, which is in line with previous studies [44]. Women win 14.3% of the trials, while men win 20.1%, and the differences are statistically significant. This can be explained because 82.1% of men request joint custody, while 86.5% of women request sole custody. Then, it must be taken into account that the justice grants the appellant individual custody 13.5% of the time and joint custody 21.4% of the time. The rulings of the Spanish Supreme Court have a significant influence on the decisions of the second instance courts and their tendency is in favor of joint physical custody. This may explain why second instance court rulings are increasingly favorable to joint physical custody. As expected, we found no statistically significant differences in the sex of the judge when granting sole or shared custody, which speaks for the neutrality of Spanish judges.

The second research question addresses whether court decisions can be predicted with a reasonable success rate. We used temporal validation; that is, the test samples come from data collected after the training data, as in a real-world application. We developed the models using logistic and neural network regression, achieving satisfactory performance measures. Our study highlights that justice is predictable in the case of child custody, which is a contribution because papers predicting judicial decisions addressed other legal concerns [13–16,30]. Holmes [11] claimed that for the rational study of the law, the man of the future must be the man of statistics, and law must be predictive. We can conclude from our analysis that it is possible to forecast court decisions in the context of child custody from a modest set of factual elements.

### Practical implications

The paper provides practical implications affecting parents, lawyers, and even the judicial system. We found statistically significant differences by considering the factual elements to be independent variables and by, rather than considering the decisions to be dependent variables, considering the requests (joint custody vs sole custody). However, interestingly, the facts influence in the opposite direction: that is, those who complain the most have the least reason to complain. This is because the courts rule in favor the plaintiff in the case of second appeal judgments on child custody only 17.8% of the time. Perhaps many parents decide to go to trial without being aware of their chances of success, leaping in the dark. We obtained useful rules for decision making using the CHAID decision tree technique. Some of the rules allow the recognition of judicial patterns with an accuracy above 90%. For example, if a couple has a very bad relationship, it is almost impossible for the judge to award joint custody (less than a 0.5% chance of winning). However, this is a very common case (about a quarter of the cases), and lawyers would probably do well to advise their client to avoid a trial under these circumstances. The practical implications of these rules are clear as they allow the preparation and filing of a lawsuit with information on the probability of success or failure. Many parents who go to court would be willing to negotiate if they knew that their chance of success is low according to the rules obtained in our empirical study. This is very much in line with the therapeutic justice process, which aims at families resolving their own disputes and encourages the use of mediators [57]. The use of legal decisional systems would make it possible to know the probabilities of success, contributing to reducing the asymmetry of information in the legal domain, which has pernicious effects [9]. If the use of these predictive models becomes widespread, there may even be effects on the judicial system. It is possible that the number of trials would decrease, and the saturation and slowness in the judicial system would be alleviated. It can also affect the work of lawyers and the way they approach a trial. The lawyer’s experience is a determining factor when it comes to winning lawsuits [58]. Studies such as the one presented can supplement experience by providing insight into the factors that judges take into account in their decisions. It could also be the case that a lawyer who wants to improve his record by winning a high percentage of cases could accept only those that a priori are more likely to be won.

### Limitations and future research

Reading and labeling 1,884 court sentences is laborious and has a certain amount of subjectivity, which is a limitation of the work. Much progress has been made in argumentation mining in the legal domain through the application of natural language processing [59]. We made several attempts to automate labeling, with qualitative text analysis software and training a neural network model for argument mining. The results were promising, and there was a high degree of agreement with the human experts, but manual labeling was chosen, because given the objective of the study, no level of discrepancy was acceptable. It is proposed as a future line of research because it would facilitate extending this type of study to other decisions in the legal domain. We identified a set of factual elements that explain court decisions, but other factual findings or even external variables, such the experience of lawyers, can be relevant as factors of success in trials [58]. It would be positive to extend the study using first instance court decisions, as we only analyzed second instance court decisions, which would increase the validity of the study. Another limitation is that the results are valid for cases in Spain; the results could be generalized to other countries, but only those with similar development indices. To overcome these limitations, it would be necessary to extend the study to other contexts, which we propose as a future line of research.
